# Controlling collective rotational patterns of magnetic rotors

**DOI:** 10.1038/s41467-019-12665-w

**Published:** 2019-10-16

**Authors:** Daiki Matsunaga, Joshua K. Hamilton, Fanlong Meng, Nick Bukin, Elizabeth L. Martin, Feodor Y. Ogrin, Julia M. Yeomans, Ramin Golestanian

**Affiliations:** 1 Rudolf Peierls Centre for Theoretical Physics, University of Oxford, Oxford, OX1 3PU UK; 2 Division of Bioengineering, Graduate School of Engineering Science, Osaka University, Toyonaka, 5608531 Japan; 3 College of Engineering, Mathematics and Physical Sciences, University of Exeter, Exeter, EX4 4QF UK; 4 QinetiQ Ltd, Cody Technology Park, Farnborough, GU14 0LX UK; 5 Max Planck Institute for Dynamics and Self-Organization (MPIDS), Göttingen, 37077 Germany

**Keywords:** Magnetic properties and materials, Fluid dynamics, Nonlinear phenomena

## Abstract

Magnetic actuation is widely used in engineering specific forms of controlled motion in microfluidic applications. A challenge, however, is how to extract different desired responses from different components in the system using the same external magnetic drive. Using experiments, simulations, and theoretical arguments, we present emergent rotational patterns in an array of identical magnetic rotors under an uniform, oscillating magnetic field. By changing the relative strength of the external field strength versus the dipolar interactions between the rotors, different collective modes are selected by the rotors. When the dipole interaction is dominant the rotors swing upwards or downwards in alternating stripes, reflecting the spin-ice symmetry of the static configuration. For larger spacings, when the external field dominates over the dipolar interactions, the rotors undergo full rotations, with different quarters of the array turning in different directions. Our work sheds light on how collective behaviour can be engineered in magnetic systems.

## Abstract (web summary)

A challenge of magnetically-actuated devices is to obtain different behaviours from each component under the same driving field. Here the authors tune the dipolar interactions between rotors to obtain different rotational behaviours when actuated by a magnetic field leading to complex collective dynamics.

## Introduction

Magnetic driving is convenient, and in recent years there have been several suggestions for ways to use magnetic colloids in microfluidic applications. These include swimmers^1–6^, pumps^7–10^, cilia^11–15^, and for particle sorting and segregation^16–19^. For reviews see refs. ^4,16,20,21^. When a uniform rotational magnetic field is applied to a ferromagnetic colloid, the colloid experiences a torque and rotates with the field. However, exploiting this to achieve a net motion or flow is not intuitive because of the low Reynolds number hydrodynamics: a single rotator in an infinite fluid produces a rotlet flow field without any pumping^22^. One way to achieve a net flow is to place the colloid near a wall, which breaks the symmetry of the rotational flow^7–9^. Another possibility is self-assembled magnetic cilia that can produce fluid flow, if the pathways of the driving and recovery strokes are different^11,12,23^.

The majority of work so far has concentrated on a single magnetic unit, or a collection of many magnetic entities that move in exactly the same way as the field rotates. To make progress in exploiting magnetic driving at low Reynolds numbers, it is of interest to devise systems where the dynamics of each unit differs, even though they receive the same external driving signal. Ideally, one would like to be able to fabricate a synthetic system that mimics the well-known natural example of ciliary arrays that have evolved to, for example, pump fluids around the body. While cilia are subject to independent individual driving, they can develop metachronal waves—phase lags between neighbours—because of hydrodynamic coupling^24,25^. Metachronal waves have been observed in magnetically driven, artificial cilia by patterning the magnetization direction^13^ or by modulating the length of the cilia^14^, and thereby engineering permanent intrinsic differences between neighbouring actuating units.

**Fig. 1:**
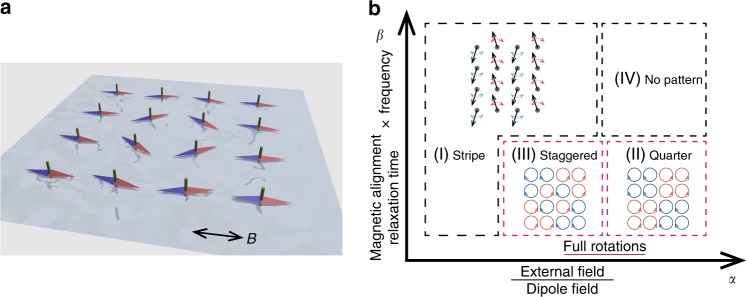
System set-up and summary of dynamical responses. **a** Schematic of the system set-up. **b** Phase diagram of the reported three collective phases as follows (see Fig. 4c for a full-phase diagram). I Stripe pattern: when the magnetic dipole interaction is dominant, the rotors swing upwards or downwards in alternating stripes. II Quarter pattern: when the external field dominates over the dipolar interactions, the rotors undergo full rotations, with different quadrants of the array turning in different directions. III Staggered pattern: when the dipole interactions and the external field are comparable, the rotors show full rotations with the rotational direction forming a staggered pattern

Here we describe the dynamics of an array of magnetic rotors (Fig. 1a) that are actuated via an oscillatory external magnetic field, using theory and experiment. We demonstrate surprisingly complex dynamics in the rotational patterns depending on the strength of the field as shown in Fig. 1b, and report three distinct collective actuation regimes, namely a stripe pattern in which the rotors swing upwards or downwards in alternating stripes, a quarter pattern in which the rotors undergo full rotations with different quadrants of the array turning in different directions, and a staggered pattern in which the rotors show full rotations with the rotational direction being staggered. We have been able to devise strategies to achieve dynamical states in which identical rotors that are actuated using the same external driving mechanism behave differently. This has been possible because of the geometry of the system, its finite size and the complex nature of magnetic dipolar interactions^26–28^. In particular, we show that the rotor array can drive an extensional flow. Our simulations help to interpret the dynamics, and show that other collective modes are also possible if the rotor array can be fabricated free of imperfections.

## Results

### Rotor array system

**Fig. 2:**
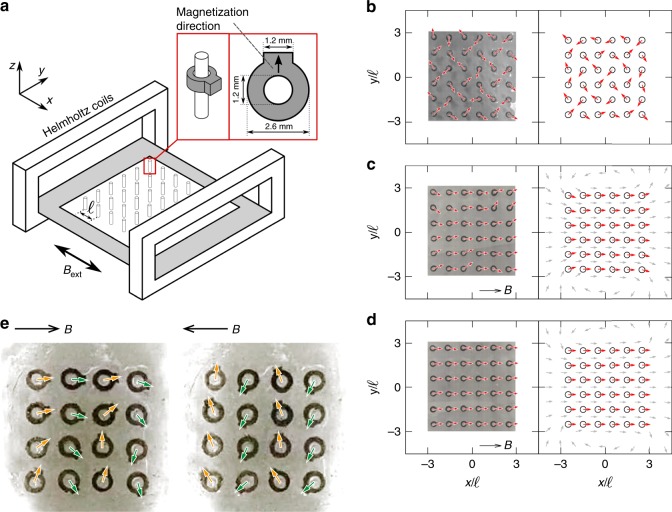
The array of rotors in static magnetic field and weak oscillating magnetic field. **a** Schematic of the experimental set-up, consisting of a Helmholtz coil system, with the 3D printed pin system placed in the centre. The inset shows a rotor mounted on a pin and indicates its dimensions. **b**–**d** Snapshots of orientational configuration under a static field with **b** $\alpha =0.0$, **c** 0.2 and **d** 10.0. The external magnetic field is imposed to right. Left column shows result from the experiment while right shows the simulation. Red arrows describe the magnetic moment direction, while grey arrows in **c**, **d** denotes magnetic field direction created by the rotors. **e** Experimental observation of the stripe swinging pattern for a 4$\times$4 rotor array under $\alpha =0.4$ and $\beta =0.3$. The arrows depict the direction of the magnetic moments, and the two frames show the moment when the external magnetic field reached $+B$ and $-B$, respectively

The system consists of $N={N}_{x}{N}_{y}$ magnetic rotors with ring geometries (Fig. 2a) positioned on a square grid, where ${N}_{x}$ and ${N}_{y}$ are the number of rotors in the $x$- and $y$-directions, respectively. The rotors are fabricated by mixing silicone rubber and NdFeB magnetic particles and they have a magnetic moment of $m=2.0\times 1{0}^{-4}{\rm{A}}\cdot {{\rm{m}}}^{2}$. As shown in an inset of Fig. 2a, the rotors have a small rectangular protrusion, which shows the magnetization direction. Details of the fabrication process are given in a subsection in Method ‘Fabrication process’.

The rotors are placed on an interface between glycerol (>95% pure; viscosity $\eta =1.4\ {\rm{Pa}}\cdot {\rm{s}}$) and air. They are held in place by a square array of 3D printed posts, where $\ell$ is the spacing between the posts. Hence, because of the posts, the rotors have no translational degree of freedom, but can freely rotate feeling the local magnetic fields. A Helmholtz coil system is used to create a uniform magnetic field along $x$ oscillating in time as:

1 $${{\boldsymbol{B}}}_{{\rm{ext}}}=(B\sin 2\pi ft,0,0),$$

where $B$ is the amplitude and $f$ is the frequency.

To write down equations of motion describing the system, we make two simplifying assumptions. First, we assume that the rotor is a sphere with radius $a$ that has no translational degree of freedom, but can freely rotate about the $z$-axis. Second, we ignore any effect of the liquid–air interface, and consider rotors located in an infinite fluid with viscosity $\eta$ and density $\rho$. Each rotor has a magnetic moment ${\boldsymbol{m}}=(m\cos \theta ,m\sin \theta ,0)$, where $\theta$ is the angle the moment makes with the $x$-axis.

A rotor $i$ experiences a magnetic torque:

2 $${T}_{i}{\hat{{\bf{e}}}}_{z}\,=\,{{\boldsymbol{m}}}_{i} \, \times \left(\,{{\boldsymbol{B}}}_{{\rm{ext}}}\,+\frac{{\mu }_{0}}{4\pi }\mathop {\sum }\limits_{j\ne i}^{N}\frac{3({{\boldsymbol{m}}}_{j}\cdot {{\boldsymbol{n}}}_{ij}){{\boldsymbol{n}}}_{ij}-{{\boldsymbol{m}}}_{j}}{{r}_{ij}^{3}}\right),$$

where ${{\boldsymbol{r}}}_{i}$ is its position vector, ${{\boldsymbol{r}}}_{ij}={{\boldsymbol{r}}}_{j}-{{\boldsymbol{r}}}_{i}$, ${r}_{ij}=| {{\boldsymbol{r}}}_{ij}|$, ${{\boldsymbol{n}}}_{ij}={{\boldsymbol{r}}}_{ij}/{r}_{ij}$ and ${\hat{{\bf{e}}}}_{z}$ is the unit vector along the $z$-direction. The first term gives the torque from the external magnetic field ${{\boldsymbol{B}}}_{{\rm{ext}}}$, while the second term describes the torque from the dipolar interactions between the rotors.

We assume Stokes flow in this system, and following Eqs. (3) and (4) are only valid for low Reynolds number regimes ${Re}\ll 1$. To the leading order in the hydrodynamic coupling between rotors, the angular velocity of a rotor $i$ is

3 $${\omega }_{i}=\frac{\mathrm{d}{\theta }_{i}}{\mathrm{d}t}=\frac{{T}_{i}}{8\pi \eta {a}^{3}}-\frac{1}{16\pi \eta }\mathop {\sum }\limits_{j\ne i}^{N}\frac{{T}_{j}}{{r}_{ij}^{3}}.$$

The coefficient in the first term $8\pi \eta {a}^{3}$ is the friction constant for the rotation of a sphere, while the second term is a consequence of the flow field produced by the rotation of the other spheres^22^. Note that the coefficient in the second term can be derived by applying a rotation operator to the rotlet flow field. Although the hydrodynamic interactions would be expected to play an important role if the rotors are in close proximity, here they only have a minor effect: the second term of Eq. (3) is two orders of magnitude smaller than the first term for the grid size in our experimental set-up, $\ell /a=5.0$.

Considering the rotors as point torques, the flow velocity ${\boldsymbol{v}}$ at position ${\boldsymbol{x}}$ can be expressed by a summation of rotlets as:

4 $${\boldsymbol{v}}({\boldsymbol{x}})=\frac{1}{8\pi \eta }\mathop {\sum }\limits_{i}^{N}\left\{\frac{1}{| {\boldsymbol{x}}-{{\boldsymbol{r}}}_{i}{| }^{3}}{\boldsymbol{T}}({{\boldsymbol{r}}}_{{\boldsymbol{i}}})\times ({\boldsymbol{x}}-{{\boldsymbol{r}}}_{i})\right\}.$$

To follow the dynamics of the array of rotors, Eqs. (2) and (3) were solved numerically. The dimensionless parameter $\tilde{a}=a/\ell =0.2$ was kept constant for all simulations.

The rotors interact with the field, and also with each other through magnetic dipole interactions. The relevant dimensionless parameters are:

5 $$\alpha =\frac{B{\ell }^{3}}{{\mu }_{0}m},$$

6 $$\beta =\frac{\eta {\ell }^{3}f}{mB}.$$

$\alpha$ is the ratio between the external magnetic field and the dipolar field due to a neighbour rotor. The second parameter, $\beta$, compares the relaxation time of the system to the external field frequency $f$. The Reynolds number, ${Re}$, is

7 $${Re}=\frac{{a}^{2}\rho f}{\eta } \sim 1{0}^{-3},$$

so inertial effects can be neglected.

We first describe the orientational patterns of the rotors when the magnetic field is static. Fig. 2b shows the configuration in zero field, $\alpha =0$. The dipole–dipole interactions maximize the angle between neighbours resulting in the frustrated spin-ice structure^29,30^. At the other limit, $\alpha \gg 1$, the dipolar forces are negligible and all the rotors align along the external magnetic field direction as shown in Fig. 2d. At an intermediate value of $\alpha \approx 1$ shown in Fig. 2c, there are small deviations from the uniform state, predominantly tilting at the corners and edges of the array: the rotors at the bottom left and top right tilt towards $+y$, while those in the other two corners show tilting towards $-y$. The local alignments are created by the magnetic field of the rotors as shown in Fig. 2c, d, and this is due to the finite size of the system.

### Collective rotational patterns

We now turn to the dynamics in an oscillatory magnetic field. The behaviour of a single rotor is shown in Supplementary Movie 1. The rotor swings backwards and forwards through $18{0}^{\circ}$ at a frequency $f$, following the field (see Fig. 1b-(I) where the pattern of rotor movement is shown). The swinging direction can be either ‘upwards’ (i.e. the polar angle $0\ < \ \theta \ < \ \pi$) or ‘downwards’ ($-\pi \ < \ \theta \ < \ 0$). The direction of swing depends only on the initial angle: if a rotor has a component of its magnetic moment along $+y$ ($-y$), it will swing upwards (downwards). This is a consequence of the small inertia condition, ${Re}\ll 1$; the rotor does not overshoot during the swinging and does not undergo a full rotation for any value of $\alpha$ or $\beta$. Despite the simplicity of the single rotor dynamics, rotor arrays have different collective dynamical modes as the control parameters are varied. Fig. 2e and Supplementary Movie 2 show the dynamics of a $4\times 4$ rotor array when an oscillatory field is applied for $\alpha =0.4$ and $\beta =0.3$ (see also Fig. 1b for the pattern of the rotor movement). Whether the spins swing upwards or downwards is controlled by the static configuration, which is the spin-ice structure. Hence, there are alternating stripes along the $y$-direction of rotors that swing up (orange arrows) or down (green arrows).

**Fig. 3:**
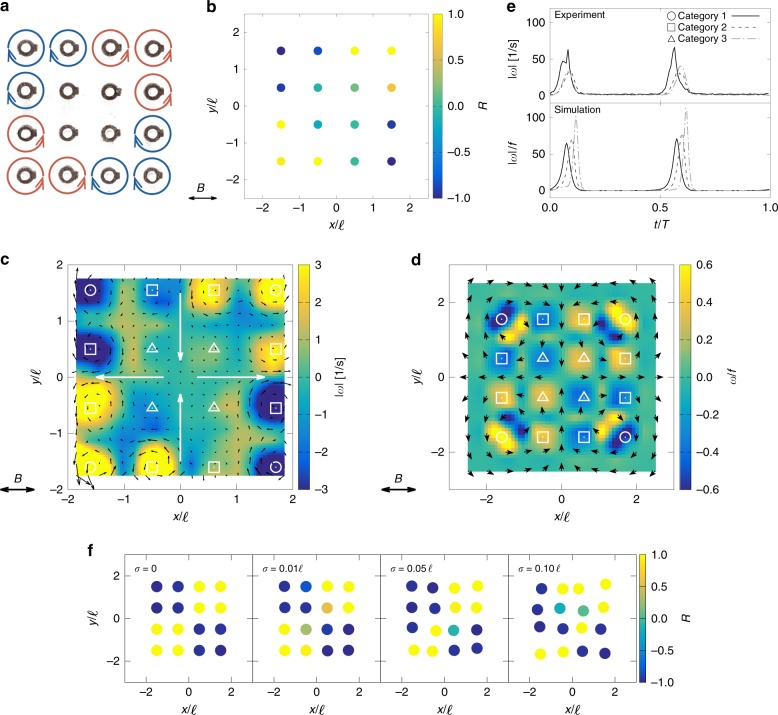
The quarter rotational pattern. **a** Schematic showing the rotational pattern under alternating magnetic field with $\alpha =6.0$ and $\beta =0.3$. **b** The rotational parameter $R$ of each rotor for a 4 × 4 rotor array. The dots depicts a single rotor, with blue meaning clockwise ($R=-1$) and yellow meaning counter-clockwise rotation ($R=1$). The observation is based on 25 cycles. **c**, **d** Time-averaged flow field generated by the rotors in **c** experiment ($\alpha =6.0$ and $\beta =0.3$) and **d** simulation ($\alpha =6.0$ and $\beta =0.03$). The contour shows the vorticity $\omega$ strength, the black arrows visualize the local flow field and the large gray arrows show net flow field created by the system. Symbols indicate the rotor position. **e** Time history of the vortex strength $| \omega |$ for three categories of rotors, in experiment and simulation. Note $T=1/f$ is the period. **f** Rotational pattern with a grid that has a Gaussian noise $\sigma$ in the rotor position. The simulation is for $\alpha =50.0$ and $\beta =0.1$

A different dynamical behaviour is observed for larger values of $\alpha$. Fig. 3a is a schematic representation of the motion of a $4\times 4$ square rotor array under the 1D alternating field with $\alpha =6.0$ and $\beta =0.3$. Supplementary Movie 3 shows the corresponding dynamics of the experimental system. There is a clear contrast to the low $\alpha$ case because the discs can now show full rotations of $2\pi$ during the cycle. This can occur because the combination of the dipolar interactions and the external field breaks the time-reversal symmetry in this system. The direction of rotation depends on the rotor position within the array: those located in the top-left and bottom-right quadrants rotate clockwise, while those in the other two corners rotate counter-clockwise. There is, however, a residual overall chiral symmetry, which is broken due to the initial conditions.

In order to characterize the pattern, we count the number of rotations during 25 cycles of the field and introduce the parameter

8 $$R=\frac{{n}_{+}-{n}_{-}}{{n}_{+}+{n}_{-}},$$

where ${n}_{+}$ and ${n}_{-}$ are the number of counter-clockwise and clockwise rotations, respectively. Fig. 3b shows the parameter $R$ for each rotor. The rotors with $| R| =1$ continuously rotate in the same direction (always clockwise corresponding to $R=1$ or counter-clockwise corresponding to $R=-1$), while they rotate evenly in both directions for $R=0$. Rotors at the corners almost always rotate in the same direction. The four central rotors, however, do not have a preferred direction and they typically swing during a cycle.

The preference for a particular rotational direction is a consequence of the dipolar forces leading to an initial tilting of the rotors near the edges of the array (see Fig. 2c). After the first half turn the alignment pattern is such that a given rotor will continue in the same sense, as long as it has sufficient time to relax towards its preferred orientation. Hence, the quarter pattern is seen for smaller values of $\beta$. This is an example showing that identical rotors, driven by the same field, can behave in different ways to each other because of collective effects.

As shown in Supplementary Movie 3 and Fig. 3d, the simulation reproduces the same quarter pattern under a condition $\alpha =6.0$ and $\beta =0.03$ (i.e. the same $\alpha$, but 10 times smaller $\beta$ than the experiment). The fact that we could only see the quarter pattern for relatively smaller values of $\beta$ in the simulation is because of the simplifications in our equations; for example, the model is not considering the effects of the liquid–air interface. The simulation result is also different in terms of the rotational patterns: all rotors are showing quarter pattern in the simulation, while the inner layer is not showing full rotation in the experiment. We believe this difference arises because of imperfections in the grid structure in the experiment. Fig. 3f shows a rotational pattern with a grid that has a small Gaussian noise (with standard deviation $\sigma$, in both the *x*- and *y*-directions) in the rotor position. Increasing the noise level, the inner layer is not showing full rotation similar to what is observed in the experiment.

To investigate how the rotational phase varies across the array we classify the rotors into three categories depending on their position as shown in Figs. 3c, d. Fig. 3e compares the time history of vorticity strength, $| \omega |$, for each category for a value of $\beta$ that isolates individual rotations. The figure indicates that the corners start to rotate first, and the phase then propagates towards the centre of the system. This phase propagation is also clearly shown in the simulations and Supplementary Movie 4. This is a finite system size effect: the rotors at the corners start to rotate first because they have the maximum tilting angle from the external magnetic field as shown in Fig. 2e. In other words, the corner rotors flip earlier than the others because they are closer to the opposite direction. This variability in the tilting angles give rise to the phase lag. This metachronal-wave-like phase propagation can be seen clearly in larger systems, such as the $20\times 20$ array shown in Supplementary Movie 5.

### Fluid mixing and pumping

This collective rotation can be used to mix or pump fluid as shown in Fig. 3c and Supplementary Movie 6. The particle image velocimetry (PIV) method is utilized to visualize the flow field. The instantaneous flow velocities close to the rotors were ~$100\,{\mathrm{mm}}\, {\mathrm{s}}^{-1}$ (corresponding to dimensionless speed $v/(\ell f)\approx 20$). As a result of the symmetry of the quarter pattern, the rotors pull the fluid into the centre from the $y$-direction, perpendicular to the external magnetic field, and push it out in $x$-direction thus creating a dipolar (or 2D extensional) flow. The dipolar flow field is reproduced by our simulations (Fig. 3d), and the flow magnitude is in the same order as in the experiment (maximum velocity ~$v/(\ell f)=30$; see also Supplementary Movie 4).

**Fig. 4:**
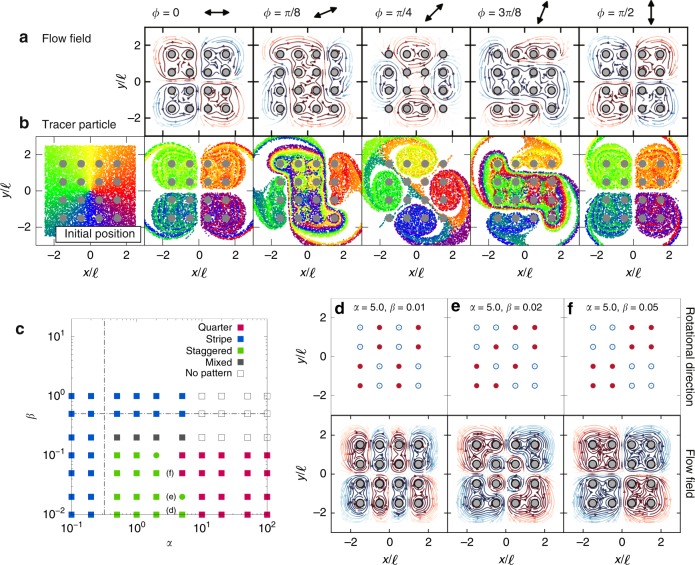
Generated flow fields and fluid mixing using the quarter and staggered patterns. **a** Generated flow field as a function of the external field direction $\phi$ for $\alpha =10.0$ and $\beta =0.05$. Streamline colours represent the vortex strength. **b** Tracer particle positions after 50 cycles of the external field actuation. **c** Phase diagram from the simulation in ${N}_{x}={N}_{y}=4$ array. Vertical dotted line shows the theoretical prediction of critical value ${\alpha }_{\mathrm{c}}=1/\pi$, while the horizontal dotted line shows the value ${\beta }_{\mathrm{s}}={\ell }^{3}/(8{\pi }^{3}{a}^{3})$. **d**–**f** Rotational patterns under three different conditions; red denotes counter-clockwise rotation, while blue denotes clockwise rotation. Bottom row shows generated flow field from the rotational patterns

Figure 4a shows rotational patterns for different external magnetic field directions defined via ${{\boldsymbol{B}}}_{{\rm{ext}}}(\phi )=B\cos 2\pi ft(\cos \phi ,\sin \phi ,0)$. When the external field is titled by 45 or 90°, it is observed that the quarter pattern also rotates by 45 and 90°. This method of creating an extensional flow has the advantage that the flow direction can be easily switched by rotating the external magnetic field. If we place this system at an intersection of microfluidic channels^10^, it can pull fluid from two directions and push it to the other two directions. At intermediate angles ($\phi =\pi /8$ or $3\pi /8$), the rotors are creating $S$-shaped vortex patterns and not showing the quarter pattern. As a demonstration, we show in Supplementary Movie 7 that the pumping can be achieved in a microchannel.

Figure 4b shows tracer particle movements after 50 cycles of actuation, whereas Supplementary Movie 8 demonstrates mixing in our experiment ($\phi =0$). By changing $\phi$, the rotors with opposite rotations determine the mixing landscape and produce drastic differences in the mixing modes. Mixing in low Reynolds number flow (or laminar flow) is challenging^31–33^, and has so far been achieved by adding polymer additives^34^ and fixed microfluidic patterning^35–37^. Our method allows us to control the mixing mode dynamically by tuning the external field, and as such introduces a practical approach for mixing fluid in low Reynolds number regimes.

### Phase diagram

We next use the simulations to explore the parameter space in more detail. Figure 4c shows which of the collective rotational patterns are stable for different values of $\alpha$ and $\beta$. For large $\alpha$ the quarter pattern is observed, as long as $\beta$ is sufficiently small, so that the system can relax to its zero field configuration between oscillations. For smaller values of $\alpha$ the stripe dynamics is stable. This is in agreement with the experiment, although the agreement is only qualitative because of the simplifications introduced in the model.

The simulations allow us to identify a range of parameters where at least two other states are stable. These, which we shall call staggered patterns, are shown in Figs. 4d, e. The rotors undergo full rotations but the pattern of clockwise and anticlockwise rotations varies. The corresponding flow fields are also shown in Figs. 4d, e. Importantly, these different rotational patterns allow us to access different length scales of fluid mixing. As shown in Fig. 3, we find that by incorporating a very small irregularity in the positions of the rotors, the simulations no longer give the same behaviour, and result in random rotational patterns. Hence, a perfect lattice structure is required to obtain these patterns. We believe this sensitivity can explain why we were unable to observe these staggered patterns experimentally.

For a comprehensive understanding of the phase diagram, we present theoretical arguments that predict the existence and approximate locations of the main phase boundaries. The magnetic dipole–dipole interaction between two rotors decays with their distance $r$ in the form of $1/{r}^{3}$; therefore, the interaction can be considered as short-ranged in our 2D system. We can thus approximate the magnetic energy of the $i$th rotor as:

9 $${U}_{i}=-{{\boldsymbol{m}}}_{i} \cdot \left({{\boldsymbol{B}}}_{{\rm{ext}}}\,+\frac{{\mu }_{0}}{4\pi }{\sum _{\langle ij\rangle} }\frac{3({{\boldsymbol{m}}}_{j}\cdot {{\boldsymbol{n}}}_{ij}){{\boldsymbol{n}}}_{ij}-{{\boldsymbol{m}}}_{j}}{{r}_{ij}^{3}}\right),$$

keeping only the magnetic dipolar interaction with the neighbouring rotors (hence the notation $\langle ij\rangle$). As shown in Figs. 2b, d, the magnetic moments of the rotors will be aligned with the magnetic field if ${{\boldsymbol{B}}}_{{\rm{ext}}}$ is strong, and they form a spin-ice structure if ${{\boldsymbol{B}}}_{{\rm{ext}}}$ is negligible. By comparing the magnetic energies of one rotor in these two configurations, we find ${\alpha }_{{\rm{c}}}=1/\pi$ (shown in Fig. 4c) as the critical value at which the two contributions match. For $\alpha \ > \ {\alpha }_{{\rm{c}}}$ the rotors will be aligned with ${{\boldsymbol{B}}}_{{\rm{ext}}}$, whereas for $\alpha \ < \ {\alpha }_{{\rm{c}}}$ the rotors will prefer the spin-ice structure.

In the phase diagram, there is a region of ‘no pattern’, where no specific patterns can be identified in the collective dynamics of the system. This behaviour originates from the fact that the rotation of the rotors in this regime is significantly slower than the oscillations of the magnetic field. This happens for large $\alpha$, where the magnetic energy due to the external magnetic field dominates the magnetic dipole–dipole interaction. Therefore, to estimate the threshold for this behaviour, we will only consider the contribution due to the external field. Then, the angular velocity of a given rotor can be simplified as:

10 $$\omega =\frac{{\mathrm{d}}\theta }{{\mathrm{d}}t}=\frac{m{B}_{{\rm{ext}}}\sin\,\theta }{8\pi \eta {a}^{3}}=\left(\frac{mB}{8\pi \eta {a}^{3}}\right)\sin\,2\pi ft \sin\,\theta .$$

This equation can be integrated to yield

11 $$\left|\tan \frac{\theta (t)}{2}\right|=\left|\tan \frac{{\theta }_{0}}{2}\right|\exp \left\{\frac{{\ell }^{3}(1-\cos 2\pi ft)}{16{\pi }^{2}{a}^{3}\beta }\right\},$$

where ${\theta }_{0}$ is the initial angle. Since the cosine function is bounded, we can recast the above solution into the following form, in terms the maximum and minimum values for the angle $\theta$ in a given cycle:

12 $$\frac{\left|\tan \frac{{\theta }_{\max }}{2}\right|}{\left|\tan \frac{{\theta }_{\min }}{2}\right|}=\exp \left\{\frac{{\ell }^{3}}{8{\pi }^{2}{a}^{3}} \cdot \frac{1}{\beta }\right\}.$$

If the above ratio remains to be around unity, the orientations of the dipoles do not change appreciably from the initial values that are randomly distributed. If the ratio becomes significantly larger than unity, the angles could deviate substantially from the initial values and merge into a collective pattern of persistent rotations. The threshold for the change in behaviour occurs at some value ${\beta }_{{\rm{s}}}={c}_{0}{\ell }^{3}/(8{\pi }^{2}{a}^{3})$, where ${c}_{0}$ is of order unity. We can also estimate the onset of ‘no pattern’ by incorporating a slipping condition: $\omega \ < \ 2\pi f$, or in other words, $\beta \ > \ \sin\,\theta \ {\ell }^{3}/(16{\pi }^{2}{a}^{3})$. We can provide an estimate of ${\beta }_{{\rm{s}}}$ by averaging the right-hand side of Eq. (3), which gives: ${\beta }_{{\rm{s}}}\simeq \frac{1}{\pi }{\int }_{0}^{\pi }\sin\,\theta {\mathrm{d}}\theta \cdot {\ell }^{3}/(16{\pi }^{2}{a}^{3})={\ell }^{3}/(8{\pi }^{3}{a}^{3})$. This estimate is shown in Fig. 4c.

### Rotational patterns for different grid configurations

**Fig. 5:**
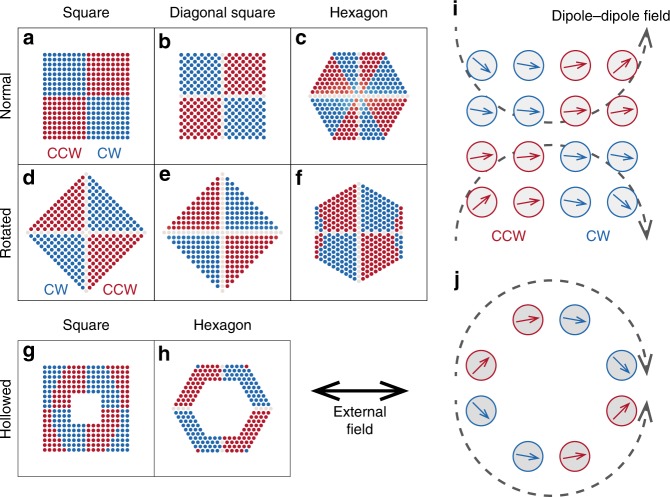
Collective rotational patterns with different grid configurations. **a**–**h** Patterns with different grid configurations; the simulations performed for $\alpha =5.0$ and $\beta =0.05$ for all configurations. Red circles represent rotors that rotate counter-clockwise, while blue circles show clockwise rotation. Gray shows a rotor that has no rotational preference. **i**, **j** Schematics showing the relation between the global grid shape and the rotational patterns

To investigate how generic our observations are, we examine the effect of the grid configuration on the collective properties of the rotors, as reported in Fig. 5. When we change the local structure from a square lattice (Fig. 5a) to a diagonal square lattice (Fig. 5b), the rotational pattern is not significantly modified. By tilting these structures by 90° (Fig. 5d, e), the position-dependent rotational pattern flips and shows counter-clockwise rotation at the bottom-right and the top-left corners. We also observe several other rotational patterns such as Fig. 5c, f–h, which contain mixed clockwise/counter-clockwise rotation patterns. These observations suggest that the local lattice structure (square or hexagonal) has a weak effect on the rotational patterns, while the overall grid shape has a larger impact.

The differences in these patterns can be traced back to the degree of roundness of the global grid boundary. As shown in the previous sections, the dipolar interactions give rise to the rotational preferences for small tilting angles (Fig. 5i). When the overall shape of the grid is rounded as in Fig. 5j, this tilting angle follows the smooth boundary since the dipole moments tend to align with their neighbours. As a result, the corners exhibit opposite rotations for Fig. 5i, j. When the global shape has both features of Fig. 5i, j, the system exhibits complex patterns as seen in Fig. 5c, f, g.

## Discussion

We have described the collective motion of magnetic rotors that are placed on a square grid. The rotors, and pins that constrain their positions, are fabricated using 3D printing technology and actuated by a uniform, oscillating magnetic field. By changing the magnetic field strength relative to the dipolar interactions between rotors we identified two different collective modes in experiments. When the magnetic dipole interaction is dominant the rotors swing upwards or downwards in alternating stripes, reflecting the spin-ice symmetry of the static configuration. For larger spacing, when the external field dominates over the dipolar interactions, the rotors undergo full rotations, with different quadrants of the array turning in different directions. This is a consequence of the dipolar perturbations to a fully aligned state, which occur because of the finite size of the array.

The motion can be used to drive an extensile flow, even at zero Reynolds number, and hence it gives a new possibility of achieving magnetic mixing or pumping at low Reynolds number. In particular, the flow direction can be easily controlled by rotating the external magnetic field.

In the quarter state different rotors have a different phase, somewhat analogous to a metachronal wave. Metachronal waves are achieved in biological cilia by hydrodynamic interactions or modulated driving. They have been demonstrated in fabricated cilia by designing the magnetic units that are intrinsically different from one another; for example, by imposing a size gradient^14^ or patterning the magnetization direction^13^. Here, by contrast, all the rotors are identical and the phase lag is an emergent property of the magnetic interactions between the dipoles as well as the geometry of the system.

We have demonstrated surprisingly complex dynamics in an array of magnetic rotors driven by an oscillating field. The results suggest several directions for further research. On the theoretical side, it will be interesting to understand how changes in the size and shape of the array affect the dynamical behaviour. Technological implementations will need to explore ways to miniaturize the devices, and the rotor configurations that will maximize the strength of the flow fields.

## Methods

### Dimensionless form of the governing equations

In this subsection, we describe the dimensionless form of the governing equations and the numerical methods. Defining the dimensionless form of the toque as ${T}^{* }=T{\ell }^{3}/({\mu }_{0}{m}^{2})$, we find the dynamical equation for the angular velocity (Eq. (3)) as:

13 $$\frac{{\omega }_{i}}{f}=\frac{1}{\alpha \beta }\left\{\frac{{T}_{i}^{* }}{8\pi {(a/\ell )}^{3}}-\frac{1}{16\pi }{\sum _{j\ne i}}\frac{{T}_{j}^{* }}{{({r}_{ij}/\ell )}^{3}}\right\}.$$

Also, the flow field given by the rotlets (Eq. (4)) is given by:

14 $$\frac{{v}_{i}}{\ell f}=\frac{1}{\alpha \beta }\ \frac{1}{8\pi {r}^{* 3}}\ {\varepsilon }_{ijk}{T}_{j}^{* }{r}_{k}^{* },$$

where $${r}^{* }=r/\ell$$.

In our simulations, the initial orientations $\theta (t=0)$ of the rotors were random, and the orientations were updated with the 1st-order Euler method with a time step $f\Delta t=1.0\, \times 1{0}^{-3}$.

### Fabrication process

The mould used to fabricate the rotors was 3D printed (Formlabs Form 2) from a design created on Autodesk AutoCAD. The liquid silicone rubber was mixed with the curing catalyst in a 10:1 ratio by weight. NdFeB powder, with an average grain diameter <10 μm, was added to the rubber mix so that it comprised ~28% of the total volume. The liquid magnetic rubber was then placed in the 3D printed mould and cured at room temperature for 6 h. The cured rotors were then placed in a Vibrating Sample Magnetometer and the magnetizing field was ramped up to 1.8 T over 17 min to saturate the rotors along the major axis of the geometry. The resulting magnetic moment was $m=2.0\, \times 1{0}^{-4}\,{\mathrm{A}}\cdot {{\mathrm{m}}}^{2}$. The rotor radius is 1.3 mm, the inner radius is 0.6 mm, and the depth is 0.9 mm.

Arrays of posts were 3D printed with different separations (4.0 and 6.3 mm). The arrays of posts were used to fix the rotors at a given position and distance from other rotors. The posts were fabricated with a radius of 0.5 mm so that the rotation of the rotors was unhindered. The system was placed within a Petri dish containing glycerol ($\eta$ = 1.4 ${\rm{Pa}}\cdot {\rm{s}}$) so that the rotors were positioned on the glycerol–air interface.

### Experimental set-up

The coil system is powered by a signal generator and power amplifier to generate the sinusoidal field, and the amplitude and frequency of the field can be varied.

The rotor motion was observed and recorded using a high-speed camera at 240 fps. The associated fluid flow field was tracked using open source PIV Software (PIVLab^38^). The PIV images were created by placing tracer particles on the surface of the fluid, and the velocities of the particles were tracked and averaged over 3000 frames of the recording. The experimental setup was designed and constructed for the project by Platform Kinetcs Ltd (https://www.platformkinetics.com/).

**Table 1:** Experimental values

Outer radius of rotors	$a=1.3$ mm
Grid size	${\ell }_{1}=4.0$, ${\ell }_{2}=6.3$ mm
Magnetic moment of rotors	$m=2.0\, \times 1{0}^{-4}$ ${\rm{A}}\cdot {{\rm{m}}}^{2}$
External magnetic field strength	${B}_{1}=1.5$, ${B}_{2}=6.0$ mT
External magnetic field frequency	$f=1.0$ Hz
Fluid viscosity	$\eta =1.4$ Pa $\cdot$ s
Fluid density	$\rho =1.3\, \times 1{0}^{3}$ ${\rm{kg}}\ {{\rm{m}}}^{-3}$
Reynolds number	${Re}=1.57\times 1{0}^{-3}$
Condition 1	$\alpha =0.4$, $\beta =0.3$
Condition 2	$\alpha =6.0$, $\beta =0.3$

Table 1 shows the experimental values that we use in our experiments.

## Supplementary information

Description of Additional Supplementary Files

Supplementary Movie 1

Supplementary Movie 2

Supplementary Movie 3

Supplementary Movie 4

Supplementary Movie 5

Supplementary Movie 6

Supplementary Movie 7

Supplementary Movie 8

## Data Availability

The data that support the findings of this study are available from the corresponding author upon reasonable request.
