# Supplementary material for: Controlling collective rotational patterns of magnetic rotors
Source: Nat Commun. 2019 Oct 16;10:4696. doi: 10.1038/s41467-019-12665-w (PMC6795886; doi:10.1038/s41467-019-12665-w)
Supplement: Supplementary file 1 — Description of Additional Supplementary Files [file 41467_2019_12665_MOESM1_ESM.pdf]

Description of Additional Supplementary Files

File name: Supplementary Movie 1

Description: Single rotor in an alternating field with amplitude $B = 6.0$ mT and frequency $f = 1.0$ Hz. This corresponds to a dimensionless parameter $\eta a^3 f/(mB) = 2.56 \times 10^{-3}$.

File name: Supplementary Movie 2

Description: The stripe pattern for $\alpha = 0.4$ and $\beta = 0.3$ for a 4×4 grid. Parameters for the simulation are $\alpha = 0.2$ and $\beta = 0.03$.

File name: Supplementary Movie 3

Description: Quadrant pattern for $\alpha = 6.0$ and $\beta = 0.3$ for a 4×4 grid. Black floats are tracer particles that are used to measure the flow field. Parameters for the simulation are $\alpha = 6.0$ and $\beta = 0.03$.

File name: Supplementary Movie 4

Description: The quarter pattern for $\alpha = 6.0$ and $\beta = 0.03$ from simulations. The movie shows the rotational pattern (left), flow velocity amplitude (middle) and vorticity magnitude (right).

File name: Supplementary Movie 5

Description: The quarter pattern for $\alpha = 6.0$ and $\beta = 0.03$ from simulations with a 20×20 grid.

File name: Supplementary Movie 6

Description: Flow field produced by the quarter pattern for $\alpha = 6.0$ and $\beta = 0.3$ for a 4×4 grid. The movie shows the rotational pattern (left), flow velocity amplitude (centre) and vorticity magnitude (right).

File name: Supplementary Movie 7

Description: Demonstration of the pumping effect. The geometry and the dimensions of the rotors are the same as in the mixing experiment. The array is placed in a circular channel, and filled with water. When activated with the AC magnetic field the flow of up to 0.2 mm per second can be achieved in a channel with 1 mm^2 cross-section. The video is played in real time, and the flow is evident from the motion of the particles (on the right hand side). The field is applied at 30 degrees with respect to the primary axes of the pattern.

File name: Supplementary Movie 8

Description: Demonstration of ink mixing using quarter rotational pattern. The system setup is as follows: the rotor size $a = 1.5 \times 10^{-4}$ [m], the grid length $\ell = 7.0 \times 10^{-4}$ [m], the magnetic moment $m = 1.76 \times 10^{-8}$ [Am^2], the external magnetic field strength $B = 8.0$ [mT], the applied frequency $f = 15$ [Hz] and glycerol is used for the fluid.
